# Relationship between social support during pregnancy and postpartum depressive state: a prospective cohort study

**DOI:** 10.1038/srep10520

**Published:** 2015-05-29

**Authors:** Mako Morikawa, Takashi Okada, Masahiko Ando, Branko Aleksic, Shohko Kunimoto, Yukako Nakamura, Chika Kubota, Yota Uno, Ai Tamaji, Norika Hayakawa, Kaori Furumura, Tomoko Shiino, Tokiko Morita, Naoko Ishikawa, Harue Ohoka, Hinako Usui, Naomi Banno, Satomi Murase, Setsuko Goto, Atsuko Kanai, Tomoko Masuda, Norio Ozaki

**Affiliations:** 1 Department of Psychiatry, Nagoya University Graduate School of Medicine, Nagoya, Japan; 2 Center for Advanced Medicine and Clinical Research, Nagoya University Hospital, Nagoya, Japan; 3 Faculty of Policy Studies, Nanzan University, Seto, Japan; 4 Liaison Medical Marunouchi, Nagoya, Japan; 5 Sugiyama Jogakuen University, Nagoya, Japan; 6 Graduate School of Education and Human Development, Nagoya University, Nagoya, Japan; 7 Graduate School of Law, Nagoya University, Nagoya, Japan

## Abstract

Although the association between social support and postpartum depression has been previously investigated, its causal relationship remains unclear. Therefore, we examined prospectively whether social support during pregnancy affected postpartum depression. Social support and depressive symptoms were assessed by Japanese version of Social Support Questionnaire (J-SSQ) and Edinburgh Postnatal Depression Scale (EPDS), among 877 pregnant women in early pregnancy and at one month postpartum. First, J-SSQ was standardized among peripartum women. The J-SSQ was found to have a two-factor structure, with Number and Satisfaction subscales, by exploratory and confirmatory factor analyses. Analysis of covariance was performed to examine how EPDS and J-SSQ scores during pregnancy affected the EPDS score at postpartum. Significant associations were found between postpartum EPDS score and both EPDS and total scores on the Number subscales during pregnancy (β = 0.488 and -0.054, ps < 0.001). Specifically, this negative correlation was stronger in depressive than non-depressive groups. Meanwhile, total score on Satisfaction subscales was not significantly associated with postpartum EPDS score. These results suggest that having a larger number of supportive persons during pregnancy helps protect against postpartum depression, and that this effect is greater in depressive than non-depressive pregnant women. This finding is expected to be vitally important in preventive interventions.

Maternal depression during pregnancy and the postpartum period affects approximately 15% of expectant mothers^1^ and 13% of new mothers within the first 6 months after delivery^2^,^3^,^4^. Due to maternal outcomes such as low quality of life^5^, high risk of suicide^6^, and dysfunctional parenting^7^, maternal depression remains one of the most serious mental health problems faced by women. It also disturbs the mother-child relationship^8^,^9^,^10^, resulting in poor outcomes of infant / child behavioral^11^, cognitive^12^,^13^,^14^, and physical health^15^,^16^. Therefore, it is important to identify predictive factors for postpartum depression.

Previous studies have investigated psychopathological or psychosocial factors that may be associated with postpartum depression. These meta-analyses suggest that the following factors are strongly or moderately correlated with postpartum depression: past history of depression (including prenatal depression); childcare stress; prenatal anxiety; and low levels of social support 4,17,18. Therefore, social support has been focused as both a factor associated with postpartum depression and a target of psychosocial intervention^19^.

The association between social support and postpartum depression has been investigated in previous studies. For example, depression scores between groups of postpartum women with either high or low levels of social support have been compared in several studies; results showed that postpartum women with low social support had significantly higher depression scores than those with high social support^20^,^21^,^22^,^23^,^24^,^25^. Other studies have examined the influence of social support on depression scores in peripartum mothers by multiple linear regression analyses. These studies showed that a depressive mood in peripartum women was associated with a perception of receiving poor support from their partners^26^,^27^,^28^,^29^. However, in these studies, depressive symptoms and perceived social support in the prepartum or postpartum periods were examined simultaneously^20^,^21^,^22^,^23^,^24^,^25^,^26^. Because social support is not always received before postpartum depression, it remains unclear whether poor social support during pregnancy is related to postpartum depression. To address this issue, it is essential to prospectively evaluate social support received during pregnancy and postpartum depressive state.

In addition, depressed women may feel more inhibited and thus communicate with fewer friends, or they may feel more pessimistic and thereby underestimate their own social support status. In such cases, they may be in a depressive state at the time of evaluation and feel as though they are receiving poor social support. However, no previous studies have considered the depressive state as a confounding factor in assessments of social support^26^,^27^,^28^,^29^. It is therefore essential to correct for perceived social support during pregnancy by taking the depressive state at the time of evaluation into account.

Previous studies have evaluated social support with various instruments. Sarason *et al.* proposed that social support could be comprehensively evaluated by two aspects of social support: 1) the number of individuals who are available to provide social support; and 2) the degree of satisfaction with the support provided, and developed the Social Support Questionnaire (SSQ). The SSQ is widely considered the most reliable self-report questionnaire for assessing these two aspects of social support^30^. Findings from a previous study using the SSQ suggested that the number of supportive persons and the satisfaction with the social support received are associated with different factors^30^; therefore, we considered these two components to be distinct concepts. To our knowledge, the effects of prepartum social support have not been examined from these two distinct aspects. We thus hypothesized that postpartum depression might be affected in different ways by the number of supportive persons and satisfaction with the level of social support received.

The Japanese version of the abbreviated SSQ (J-SSQ) has demonstrated reliability and validity among normal and psychiatric populations^31^. However, it remains unclear whether the J-SSQ is appropriate for women in peripartum periods. Therefore, before the relationship between social support in pregnancy and postpartum depression is examined, the J-SSQ must be standardized among peripartum women.

Therefore, the aims of our study were as follows: 1) to examine the reliability and validity of the J-SSQ among peripartum women; and 2) to reveal whether postpartum depression is associated with the number of individuals who are available to provide social support and/or the degree of satisfaction with the level of social support received during pregnancy.

## Results

### Descriptive statistics

A total of 912 Japanese women gave their consent to participate in this study. They were asked to complete the J-SSQ and the Edinburgh Postnatal Depression Scale (EPDS), a self-report questionnaire used to assess pregnancy and postpartum depression, at an early stage of pregnancy (T1: before the 25^th^ week of gestation) and at one month after childbirth (T2). Among these 912 women, 888 (97.4%; mean age ± standard deviation [SD] = 32.2 ± 4.4) completed the J-SSQ in full at T1. These data were used to perform factor analyses and calculate the internal consistency of the J-SSQ.

To investigate the relationship between J-SSQ and EPDS scores at T1 and T2, we examined the data of 877 participants (mean age ± SD = 32.2 ± 4.4) who completed both the J-SSQ and EPDS in full at T1, and 371 participants (mean age ± SD = 32.5 ± 4.4) who completed the J-SSQ and EPDS in full at both T1 and T2. The numbers of the subjects who completed both the J-SSQ and the EPDS differed in T1 (n = 877) and T2 (n = 371) because when this cohort study began, the J-SSQ was not administered at T2. A more detailed explanation can be found in our previous report^32^.

Descriptive statistics were calculated for the 12 items on the J-SSQ as shown in Table 1. The scores on the Number subscale were log-transformed for analysis due to skewed distributions.

### Factor analyses of the J-SSQ items

For the purpose of factor analyses, only data from the 888 subjects who answered all the J-SSQ items at T1 were used. We randomly divided the subjects into two groups (Group 1, n = 447; Group 2, n = 441) to conduct an exploratory factor analysis (EFA) of the 12 items on the J-SSQ. The J-SSQ is composed of 12 items divided into two subscales. The first subscale (Number subscale) assesses the number of persons supporting the subject in six separate situations. The second subscale (Satisfaction subscale) rates the subject’s satisfaction with the social support they receive in each of those situations, ranging from very dissatisfied to very satisfied (1-6). For Group 1, log-transformed scores on the Number subscale and scores on the Satisfaction subscale of the J-SSQ were then subjected to EFA. The results are shown in Table 1. Based on the scree test, two factors were extracted. The first factor was loaded by the six items of the Number subscale, while the second factor was loaded by the six items of the Satisfaction subscale. We defined the sum of six items of Number subscale of the J-SSQ as “Number of Persons” (NP), and the sum of six items of Satisfaction subscale of the J-SSQ as “Satisfaction Rating” (SR). A modest correlation (r = 0.19) was observed between NP and SR.

**Figure 1:**
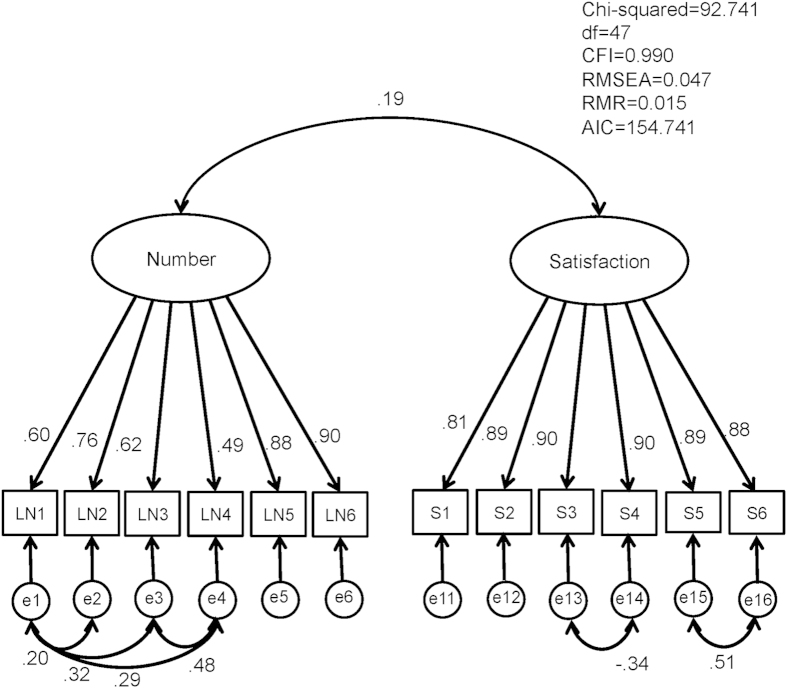
Confirmatory factor analysis of J-SSQ scores at T1 in half of the sample (Group 2, n = 441) df, degrees of freedom; CFI, comparative fit index; RMSEA, root mean square error of approximation; RMR, root mean square residual; AIC, Akaike Information Criterion; LN, log-transformed score on each item of the Number subscale of the J-SSQ; S, score on each item of the Satisfaction subscale of the J-SSQ.

Confirmatory factor analysis (CFA) was conducted to test the factor model identified by EFA using the combined data of Group 2. The model derived from EFA indicated good fit to the data; chi-square divided by degrees of freedom [CMIN/df] = 1.973; comparative fit index [CFI] = 0.990; root mean square error of approximation [RMSEA] = 0.047; root mean square residual [RMR] = 0.015; Akaike Information Criterion [AIC] = 154.741 (Fig. 1). The two latent factors were nearly uncorrelated. We also tested the one-factor model, which did not show goodness of fit with our data (CMIN/df = 6.912; CFI = 0.947; RMSEA = 0.116; RMR = 0.035; AIC = 347.568).

### Internal consistency of the J-SSQ

For the combined data of Group 1, internal consistency (Cronbach’s alpha) of the six Number items was 0.898, while that of the six Satisfaction items was 0.961, showing high internal consistency.

### Convergent validity of the J-SSQ

The EPDS scores were significantly negatively correlated with both NPs and SRs at T1 (r = −0.138 and −0.243, respectively, both p < 0.01). This was also true at T2 (r = −0.255 and −0.197, respectively, both p < 0.01).

### Difference in J-SSQ scores between the depressive and non-depressive groups at T1 and T2

NPs and SRs of the non-depressive group were significantly higher at both T1 and T2 than those of the depressive group (Tables 2 and 3, respectively). The depressive group reported significantly lower social support (both NPs and SRs) than the non-depressive group at both T1 and T2 (NP at T1, p < 0.001; SR at T1, p = 0.003; NP at T2 < 0.001; and SR at T2 p = 0.001).

### Multivariate linear regression analysis for the influence of social support during pregnancy on postpartum depression

Based on multiple linear regression analysis, NP was significantly negatively associated with EPDS score at T2 (ß = −0.054, p < 0.001). In addition, both EPDS score at T1 and primiparity were also significantly positively associated with EPDS score at T2 (ß = 0.488, p < 0.001 and ß = 2.295, p < 0.001, respectively) (Table 4).

On the other hand, maternal age and satisfaction with social support received during pregnancy were not significantly associated with the maternal postpartum depressive symptomatology identified by EPDS.

### Analysis of covariance to investigate the influence of pregnant depression and NP on postpartum depression

To assess the homogeneity protective effect of NP on postpartum depressive state, we conducted an analysis of covariance (ANCOVA) including prepartum depression, NP, and the interaction term of the two variables.

A significant interaction was found between prepartum depressive state and NP by ANCOVA (p = 0.015). In the non-depressive group, the coefficient of NP during pregnancy reducing the postpartum EPDS score was −0.046 (p = 0.003), while that in the depressive group was −0.181 (p = 0.001), suggesting that the favorable effect of NP during pregnancy was significantly stronger in the depressive than in the non-depressive group (Table 5).

## Discussion

To the best of our knowledge, the present study is the first to investigate whether social support during pregnancy influences postpartum depression in a large cohort of pregnant women.

The reliability and validity of the J-SSQ were examined among the subjects during the peripartum period. The two-factor structure (i.e., number of available supportive persons and satisfaction with level of social support received) was confirmed in this cohort. The internal consistencies of the two factors were highly satisfactory. In addition, the two subscales of the J-SSQ correlated negatively with EPDS scores both during pregnancy and at one month after delivery. This is consistent with the results of previous studies^4^,^17^,^18^,^33^,^34^, and demonstrates that the J-SSQ has acceptable convergent validity. The J-SSQ is therefore considered to be a reasonable scale for evaluating the level of social support received by women during the peripartum period.

Social support in the prepartum and postpartum periods were compared between women with and without depression. The women with both prepartum and postpartum depression reported having fewer available supportive persons and lower levels of satisfaction with the social support they received than the non-depressed women. The results of the present study correspond to those of previous reports^23^,^24^,^28^, except for one study that was conducted with a much smaller sample (n = 80)^25^. These cross-sectional comparisons did not reveal whether receiving less social support during pregnancy was related to postpartum depression. Therefore, we prospectively investigated whether prepartum social support had an effect on postpartum depressive state.

Multiple regression analysis, corrected by maternal age, showed that postpartum depression was significantly associated with prepartum depressed mood, parity, and available number of persons who could provide support during pregnancy; however, satisfaction with support during pregnancy was not significantly associated with postpartum depressive state.

The association observed between prepartum and postpartum depressed mood was consistent with results from previous studies^4^,^17^,^18^,^25^,^28^,^35^.

We also clarified the relationship between postpartum depressive state and available amount of social support received during pregnancy. We believe the present study to be the first to demonstrate that fewer supportive persons during pregnancy is a predictor for postpartum depression. This finding points to the benefits of early intervention to increase the number of support providers for pregnant women^19^.

Satisfaction with social support during pregnancy was not significantly associated with maternal postpartum depression. A previous study examined prospectively whether satisfaction with social support during pregnancy affected postpartum depression^28^. That study used the Multidimensional Scale of Perceived Social Support (MSPSS) to evaluate pregnant women’s level of satisfaction with social support from their family, friends, and significant others, independently. Logistic regression analysis demonstrated that the MSPSS scores regarding support from each group were not significantly related to postpartum depression. In the present study, treating prepartum depression as a covariate, we also performed logistic regression analysis; no significant relationship was found (odds ratio = 0.986; 95% confidence interval = 0.935–1.048; p = 0.535). These results demonstrated that, regardless of treating depression as a covariate, satisfaction with social support was not significantly related to postpartum depression.

Our results suggest a discrepancy between the number of supportive persons and satisfaction with social support in relation to postpartum depression. A modest correlation (r = 0.19) was observed between the sum of Number and Satisfaction subscales. This discrepancy is in line with the findings by Sarason’s study^30^, indicating that it reflects two different aspects of social support. Sarason *et al.* also suggested that this divergence could be influenced by the personality traits of the subjects. Personality traits will thus need to be examined as covariates in a future study.

Based on ANCOVA, the number of supportive persons during pregnancy has a stronger effect on decreasing postpartum depressive symptomatology in depressed than in non-depressed mothers. No previous studies have reported any such differences in relation to the effect of social support. This result suggests that intervention utilizing a social network is more effective for pregnant mothers with depressive symptoms, and supports the clinical importance of psychosocial interventions for depressed mothers during pregnancy.

Our results also showed that primiparous women have a higher risk for postpartum depression than multiparous women, which is inconsistent with results from previous studies^36^,^37^. For example, one study compared depression between multiparous and primiparous women, and showed that depression was significantly more prevalent in multiparous women^36^. In another study, mean postpartum EPDS scores did not significantly differ in parity between primiparous and multiparous women^37^. This result may have been due not only to methodology, but also to cultural and other psychosocial background factors surrounding pregnant women. Our study considered social support status as a covariate. Cultural and other psychosocial factors need to be treated as covariates in future studies.

Recent neuroimaging studies have also been conducted to investigate the correlation between perceived social support and monoamine activity in the brain. Single-photon emission computed tomography (SPECT) was used to examine serotonin transporter (SERT) availability in healthy volunteers; results showed that SERT availability was significantly associated with perceived social support in males, but not in females^38^. Another SPECT study in male and female healthy volunteers examined dopamine transporter (DAT) availability; results showed that DAT availability was also significantly related to perceived social support^39^. In addition, study using positron emission tomography reported observing a positive correlation between dopamine D2/3 receptor binding potential and both perceived social support and social status^40^. These studies suggest that the monoamine system may be responsible for the favorable effect on postpartum depression associated with the number of persons providing social support. More neuroimaging studies are needed to confirm the neurobiological basis underpinning our results.

This study did have some limitations. First, we defined depressive state using an EPDS cut-off score, and the diagnostic interviews were not administered by psychiatrists. Our results would be of greater value if the diagnoses had been made by psychiatrists. Second, numerous factors are known to be associated with postpartum depression^4^,^17^,^18^; however, we could not investigate all of these factors in this study.

In summary, our results indicate that fewer available supportive persons during pregnancy is a predictive factor of postpartum depression. Having a larger number of people available to provide social support during pregnancy has a greater protective effect on pregnant mothers with than without depression. These results suggest that psychosocial interventions that focus on the social support network are effective in preventing postpartum depression, especially for mothers with depression during pregnancy.

## Methods

### Participants

Participants in this study were recruited from among those participating in a prepartum program during pregnancy (starting before the 25^th^ week) at two obstetrical hospitals and one university hospital between August 2004 and June 2014. All three hospitals were located in Nagoya, an urban area in central Japan with a population of approximately 2 million people. Mothers with current or a previous history of mental illness were excluded, as were mothers with children born before the 32^nd^ week of gestation. In addition, participants were required to be at least 20 years old and capable of understanding the Japanese language.

### Measures

J-SSQ: The J-SSQ, which was standardized by Furukawa *et al.*, is the Japanese version of the SSQ6^31^. The SSQ6^41^ is a brief measure of social support based on the long form (27 items) of the SSQ^30^. The SSQ and SSQ6 are highly related and have excellent reliability and validity. The SSQ6 is composed of 12 items divided into two subscales. The first subscale (Number subscale) assesses the number of supportive persons. The second subscale (Satisfaction subscale) rates the subject’s satisfaction with the social support they receive, ranging from very dissatisfied to very satisfied (1-6), based on how many persons are reliable sources of support in each of the following six situations: 1) is dependable when you need help; 2) helps you feel relaxed when you are under pressure; 3) takes care of you regardless of the circumstances; 4) cheers you up when you are feeling down; 5) consoles you when you are upset; and 6) accepts you unconditionally, including both your good and bad points.

The total number of persons listed and the satisfaction rating for the six items constitute NP and SR, respectively. NP is the sum of the perceived number of available others who provide social support as measured by the total score on the Number subscale of the J-SSQ. SR reflects the sum of the individual’s degree of satisfaction with the perceived support available in each situation, as measured by the total score on the Satisfaction subscale of the J-SSQ.

EPDS: The EPDS is a self-reported questionnaire designed to assess pregnancy and postpartum depressive state^42^. Each item is scored on a four-point Likert scale (0-3). This instrument has been used during the prepartum and/or postpartum period^43^. The Japanese version of the EPDS showed good internal consistency (Cronbach’s alpha = 0.78) and test-retest reliability (Spearman’s correlation coefficient = 0.92)^44^. A cut-off score with a threshold of 8/9 was found to indicate pregnancy and postpartum depression among Japanese women with a specificity of 93% and a sensitivity of 75%^44^.

### Study Design

Pregnant women attending a prepartum program were given detailed information about the study orally and on paper. Women who agreed to cooperate in the study were asked to complete self-report questionnaires, which included questions on social demographics, the EPDS, and the J-SSQ in the early stage of pregnancy (T1: before the 25^th^ week of gestation), and return them by post. After receiving the completed consent forms and questionnaires, the EPDS and the J-SSQ questionnaires were sent again one month after childbirth (T2) and asked to be returned by post. Our sampling schedule was described in detail in previous reports^32^,^45^,^46^.

Depression and perceived support were assessed at both T1 and T2. Depressive symptoms and social support were measured using the EPDS and the J-SSQ, respectively.

### Statistical analysis

We calculated descriptive statistics for the J-SSQ. We log-transformed the scores for all items that were positively skewed. The participants were randomly divided into two groups (Group 1, n = 447; Group 2, n = 441). Student’s t-tests were conducted for age and EPDS scores between Group 1 (mean age ± SD, 32.2 ± 4.4; mean EPDS scores ± SD at T1, 4.7 ± 4.2; at T2, 4.8 ± 4.3) and Group 2 (mean age, 32.1 ± 4.5; mean EPDS scores at T1, 4.8 ± 4.6; at T2, 5.3 ± 4.4). No significant differences were found between the two groups in age or EPDS scores at T1 or T2. We conducted EFA of the 12 items of the J-SSQ at T1 using the data from Group 1. All factors were considered dependent upon each other, and thus a factor solution was attempted after an oblique Promax rotation. The number of factors was determined by the scree test^47^. To create a subscale of the J-SSQ, we extracted items for each subscale if they were loaded greater than 0.5 on particular factors, but less than 0.5 on other factors.

The factor structure derived from EFA was confirmed by CFA in the other half of the subjects (Group 2). The fit of each model with the data was examined in terms of CMIN/df, CFI, RMSEA, and RMR. According to conventional criteria, a good fit is indicated by CMIN/df < 2, CFI>0.97, RMSEA < 0.05, and RMR < 0.08^48^. AIC was used to compare different models; a model with an AIC value at least 2 points lower than that of a competing model is regarded as a better model.

We calculated subscale scores by adding all the item scores belonging to each factor. For all analyses except the EFA and CFA, we used raw scores on the J-SSQ to account for convenience of clinical use. Alpha coefficients for the two hypothesized subscales were calculated to examine the internal reliability of the J-SSQ.

We also constructed a correlation matrix with the EPDS. This analysis provided evidence of convergent validity. Correlation between EPDS scores and NP or SR at T1 or T2 was examined using Spearman’s correlation coefficients.

Subjects were then classified into two groups: the non-depressive group (EPDS score  < 9) or the depressive group (EPDS score ≥9), and J-SSQ scores at T1 and T2, respectively, were compared using the Mann-Whitney U test.

To examine whether the prepartum J-SSQ score was an independent predictor for the postpartum EPDS score, we performed multiple linear regression analysis with age at the time of delivery, parity, EPDS score at T1, and NP and SR at T1 as covariates.

To investigate whether the prepartum J-SSQ score was a predictor for postpartum depression, we also performed multivariate logistic regression analysis with EPDS score at T1, NP and SR at T1, and parity as covariates. In this analysis, the regression model was adjusted for maternal age.

To assess the homogeneity protective effect of NP on postpartum depressive state, we conducted ANCOVA including prenpartum depression, NP, and the interaction term of the two variables. This regression model was also corrected by maternal age.

All statistical analyses were performed using SPSS version 22.0 (IBM Japan, Tokyo, Japan) and Amos 19.0 (IBM Japan).

## Ethics Statement

The study was described to all participants both verbally and in writing, and written informed consent was obtained from each participant. This study protocol was approved by the Ethics Committees of the Nagoya University Graduate School of Medicine and other participating institutes and hospitals. The study was conducted in accordance with the established ethical standards of all institutions.

## Additional Information

**How to cite this article**: Morikawa, M. *et al.* Relationship between social support during pregnancy and postpartum depressive state: a prospective cohort study. *Sci. Rep.* **5**, 10520; doi: 10.1038/srep10520 (2015).

## Figures and Tables

**Table 1:** Factor structure of J-SSQ items during early pregnancy (Group 1, n = 447).

**Item No.**	**Factor 1**	**Factor 2**	**Range**	**Mean (SD)**	**Skew**	**Skew after log transformation**
Number*						
5	**0.888**	−0.005	0-30	3.61 (2.66)	2.41	−0.007
6	**0.861**	−0.015	0-30	3.51 (2.53)	2.70	0.063
2	**0.752**	−0.008	0-30	3.00 (2.25)	3.38	0.134
3	**0.734**	0.006	0-30	4.28 (2.71)	2.72	0.020
1	**0.678**	0.019	0-30	4.59 (2.45)	2.36	−0.121
4	**0.633**	0.005	1-50	4.75 (3.08)	5.39	0.237

Satisfaction						
6	−0.016	**0.941**	1-6	4.99 (1.13)	−1.845	—
5	0.022	**0.933**	1-6	4.99 (1.13)	−1.803	—
4	0.007	**0.923**	1-6	5.08 (1.10)	−2.049	—
2	0.026	**0.907**	1-6	4.94 (1.14)	−1.737	—
3	0.012	**0.898**	1-6	5.05(1.11)	−1.989	—
1	−0.047	**0.850**	1-6	4.95 (1.18)	−1.872	—

J-SSQ, Japanese version of the abbreviated Social Support Questionnaire; SD, standard deviation.

Bolded values are factor loadings >0.5 on one factor and <0.5 on another factor.

^*^Each item score on the Number subscale was log-transformed to reduce skew.

**Table 2:** J-SSQ scores of non-depressive and depressive groups at T1.

	**Non-depressive group (n = 303)**		**Depressive group (n = 68)**		**p-value**
	**Median**	**IQR**	**Median**	**IQR**	
NP	22	16.00-30.00	16	12.00-25.00	<0.001
SR	31	30.00-35.00	30	26.25-33.00	0.003

Mann-Whitney test.

Non-depressive group: EPDS scores <9; Depressive group: EPDS scores ≥9.

NP: total score on the Number subscale in the J-SSQ; SR: total score on the Satisfaction subscale in the J-SSQ.

J-SSQ, Japanese version of the abbreviated Social Support Questionnaire; T1, before the 25^th^ week of gestation; IQR, interquartile range; EPDS, Edinburgh Postnatal Depression Scale.

**Table 3:** J-SSQ scores of non-depressive and depressive groups at T2.

	Non-depressive group (n = 285)		Depressive group (n = 86)		p-value
	Median	IQR	Median	IQR	
NP	22	17.00-30.00	17	13.00-23.00	<0.001
SR	30	29.00-36.00	30	26.75-32.00	0.001

Mann-Whitney test.

Non-depressive group: EPDS scores <9.

Depressive group: EPDS scores ≥9.

NP: total score on the Number subscale in the J-SSQ.

SR: total score on the Satisfaction subscale in the J-SSQ.

J-SSQ, Japanese version of the abbreviated Social Support Questionnaire; T2, 1 month after childbirth; IQR, interquartile range; EPDS, Edinburgh Postnatal Depression Scale.

**Table 4:** Multiple linear regression analysis of EPDS scores at T2.

	**Coefficient (β)**	**SE**	**p-value**
Maternal age	0.031	0.048	0.515
EPDS score at T1	0.488	0.048	<0.001
NP at T1	−0.054	0.015	<0.001
SR at T1	−0.024	0.064	0.474
Primiparous	2.295	0.544	<0.001

Dependent variable: EPDS scores at T2.

NP: total score on the Number subscales in the J-SSQ.

SR: total score on the Satisfaction subscales in the J-SSQ.

EPDS, Edinburgh Postnatal Depression Scale; T2, 1 month after childbirth; T1, before the 25^th^ week of gestation; SE, standard error; J-SSQ, Japanese version of the abbreviated Social Support Questionnaire.

**Table 5:** ANCOVA results for EPDS scores at T2.

	**Coefficient (β)**	**SE**	**p-value**
Maternal age	−0.003	0.050	0.946
Depression at T1	7.228	1.211	<0.001
NP at T1	−0.046	0.015	0.003
Depression at T1 × NP at T1	−0.242	0.055	0.015
SR at T1	−0.135	0.034	0.158
Primiparous	2.232	0.565	<0.001

NP: total score on the Number subscales in the J-SSQ.

SR: total score on the Satisfaction subscales in the J-SSQ.

Depression: EPDS score ≥9.

ANCOVA, analysis of covariance; EPDS, Edinburgh Postnatal Depression Scale; T2, 1 month after childbirth; T1, before the 25^th^ week of gestation; SE, standard error; J-SSQ, Japanese version of the abbreviated Social Support Questionnaire.
